# The *A* Allele at rs13419896 of *EPAS1* Is Associated with Enhanced Expression and Poor Prognosis for Non-Small Cell Lung Cancer

**DOI:** 10.1371/journal.pone.0134496

**Published:** 2015-08-11

**Authors:** Andika C. Putra, Hidetaka Eguchi, Kian Leong Lee, Yuko Yamane, Ewita Gustine, Takeshi Isobe, Masahiko Nishiyama, Keiko Hiyama, Lorenz Poellinger, Keiji Tanimoto

**Affiliations:** 1 Department of Radiation Medicine, Research Institute for Radiation Biology and Medicine, Hiroshima University, Hiroshima, Japan; 2 Division of Translational Research, Research Center for Genomic Medicine, Saitama Medical University, Saitama, Japan; 3 Cancer Science Institute of Singapore, National University of Singapore, Singapore, Singapore; 4 Division of Clinical Oncology and Respiratory Medicine, Department of Internal Medicine, School of Medicine, Shimane University, Shimane, Japan; 5 Department of Molecular and Cellular Pharmacology, Graduate School of Medicine, Gunma University, Gunma, Japan; 6 Natural Science Center for Basic Research and Development, Hiroshima University, Hiroshima, Japan; 7 Department of Cell and Molecular Biology, Medical Nobel Institute, Karolinska Institute, Stockholm, Sweden; University of Tennessee Health Science Center, UNITED STATES

## Abstract

Hypoxia-inducible factor-2α (HIF-2α, or EPAS1) is important for cancer progression, and is a putative biomarker for poor prognosis for non-small cell lung cancer (NSCLC). However, molecular mechanisms underlying the EPAS1 overexpression are not still fully understood. We explored a role of a single nucleotide polymorphism (SNP), rs13419896 located within intron 1 of the *EPAS1* gene in regulation of its expression. Bioinformatic analyses suggested that a region including the rs13419896 SNP plays a role in regulation of the *EPAS1* gene expression and the SNP alters the binding activity of transcription factors. *In vitro* analyses demonstrated that a fragment containing the SNP locus function as a regulatory region and that a fragment with *A* allele showed higher transactivation activity than one with *G*, especially in the presence of overexpressed c-Fos or c-Jun. Moreover, NSCLC patients with the *A* allele showed poorer prognosis than those with *G* at the SNP even after adjustment with various variables. In conclusion, the genetic polymorphism of the *EPAS1* gene may lead to variation of its gene expression levels to drive progression of the cancer and serve as a prognostic marker for NSCLC.

## Introduction

Hypoxia-inducible factors (HIFs) are heterodimeric transcription factors that are members of the Per-ARNT-Sim (PAS) family. They are activated by a number of signaling inputs including hypoxia, nutrient starvation, inflammation, oncogenic signals, mechanical stress, and in some cases, internal genetic polymorphisms [1–4]. The HIF transcription factors consist of alpha subunits (HIF-1α, HIF-2α, HIF-3α) that are regulated by the aforementioned signals while beta subunits known as aryl hydrocarbon receptor nuclear translocator (ARNT) are constitutively expressed and stimulate the transcription of more than a hundred target genes related to patho-physiological response [5, 6]. Among alpha subunits, HIF-1α and HIF-2α have been extensively studied. Although members of the alpha subunits share similarities in their structure, function and regulation *in vitro*, their roles *in vivo* are disparate during development and tumorigenesis [3, 5, 7].

The human HIF-2α gene known as endothelial PAS domain protein 1 (*EPAS1*), contains 16 exons and spans 90 kb on 2p21-p16. Expression of human HIF-2α has been identified in lung, carotid bodies, endothelial cells, glial cells, cardiomyocytes, renal fibroblasts and hepatocytes where it plays an important role in the regulation of oxygen physiology [3, 8]. This is of particular importance in the lung as it constitutes the site for oxygen exchange and provides the air-liquid interface for this purpose. HIF-2α proteins are expressed in type II pneumocytes and pulmonary endothelial cells in response to hypoxia as well as in epithelium and mesenchymal structures that give rise to the vascular endothelium [9, 10]. Furthermore, high levels of HIF-2α expression were linked to increased tumor size, invasion and angiogenesis in murine models of lung cancer [11,12]. Enhanced expression of HIF-2α protein in non-small cell lung cancer (NSCLC) tissue was reported to be a significant maker for poor prognosis [13–15].

Recently, it has been reported that several single nucleotide polymorphisms (SNPs) of *EPAS1* are associated with the development of osteoarthritis [16], retinopathy of prematurity [17], maximum metabolic performance in elite endurance athletes [18], physiologic adaptation in high altitude populations [19–22], and susceptibility towards renal cell carcinoma (RCC) and prostate cancer [23, 24]. However, the effects of these SNPs on expression levels of the *EPAS1* are scarcely understood.

Among these SNPs, we focused on Hap-tag SNPs of the *EPAS1* gene that may contribute to the adaptation to high-altitude hypoxia in Sherpas [22], considering lung as a target organ. Bioinformatic analyses prompted us to examine the role of rs13419896 SNP in regulation of the *EPAS1* gene expression and an association with prognosis of NSCLC.

## Materials and Methods

### Bioinformatic analyses

We interrogated transcription factor chromatin immunoprecipitation (ChIP-seq) datasets from the Encyclopedia of DNA Elements (ENCODE) consortium using the University of California, Santa Cruz (UCSC) Genome Browser (http://genome.ucsc.edu/ENCODE/) to find out candidate transcription factors that may bind on or in close proximity to the rs13419896 SNP site. Allele specific surveillance of transcription factors bound to the fragment containing the rs13419896 SNP was carried out using JASPAR CORE Vertebrata, an open-access database of matrix-based nucleotide profiles describing the binding preference of transcription factors [25].

### DNA extraction and genotyping analysis

Genomic DNA was isolated from peripheral blood samples or frozen non-cancerous lung tissues as previously described [26, 27]. The following primer set was used to amplify a fragment including the SNP focus in *EPAS1* intron1; Forward: 5’-CCTAATGAGCCTCTGGGAAAGTGC-3’ and Reverse: 5’-CAATGGTGCCTCCTACCCTGTG-3’. The PCR reaction conditions were 40 cycles of denaturation at 95°C for 30 sec, annealing at 63°C for 30 sec, and extension at 72°C for 30 sec. Sequencing of PCR products was carried out using the BigDye Terminator Cycle Sequencing Kit and ABI PRISM 310 Genetic Analyzer automated sequencing system (Applied Biosystems, Foster City, CA, USA).

### Cell lines and cell culture

Twenty-four human cancer cell lines consisting of a hepatoma cells HepG2, oral squamous cell carcinoma lines HSC-2, HSC-3, HSC-4, KB and KOSC2, breast cancers MCF-7, MDA-MB-231, MDA-MB-435S, MDA-MB-453, MDA-MB-468, BT-20, BT-474, SK-BR-3, T-47D, and ZR-75-1 and lung cancer cell lines A549, PC-6, PC-9, PC14, RERF-LC-Ad-1, RERF-LC-Ad-2, RERF-LC-KJ, and LC-S were obtained from ATCC (Manassas, VA) or JCRB (Osaka, Japan) between 2001 and 2007, and maintained in RPMI1640 or Dulbecco’s modified Eagle’s minimal essential medium (DMEM) (NACALAI TESQUE, Inc., Kyoto, Japan) containing 10% fetal bovine serum (FBS; BioWhittaker, Verviers, Belgium) as previously described [28–30]. *EPAS1* status of the cells was determined by sequencing analysis as described above.

### RNA preparation and RT-PCR

Total RNA was prepared from frozen cell pellets using the QIAGEN RNeasy mini kit (QIAGEN, Inc., Valencia, CA) according to manufacturer instructions. Two micrograms of total RNA extracted from each cell line was reverse-transcribed using the High-Capacity cDNA Archive Kit (Applied Biosystems, Foster City, CA). A 1/200 dilution of the cDNA was subjected to real-time RT-PCR using TaqMan Gene Expression Assays (Applied Biosystems) for *EPAS1* and Pre-Developed TaqMan Assay Reagents (Applied Biosystems) for *ACTB* as housekeeping control. Three independent measurements were taken and averaged with relative gene expression levels calculated as ratios over *ACTB* expression for each cell line.

### Immunoblot Analysis

To analyze protein expression, whole cell extracts were prepared from cultured cells with or without hypoxic treatment as previously described [30]. Fifty μg of protein was blotted onto nitrocellulose filters following SDS-polyacrylamide gel electrophoresis. Anti-EPAS1 (HIF-2α) (Cell Signaling Technology Japan, Tokyo) or anti-β-actin (sigma) was used as primary antibodies diluted as 1:500 or 1:5000. Anti-rabbit IgG or anti-mouse Ig horseradish peroxidase conjugate (Amersham Life Science) was used as a secondary antibody diluted as 1:2000 or 1:5000. Immunocomplexes were visualized using the enhanced chemiluminiscence reagent ECL Plus (Amersham Life Science).

### Plasmid construction and luciferase reporter experiments

Annealed oligonucleotide fragments containing the *EPAS1* SNP locus rs13419896 (5’-GGTACCAGTGTCTGAAAGTGAAGC**G**CTAGGATTGGTTACTGACGGTACC-3’ or 5’-GGTACCAGTGTCTGAAAGTGAAGC**A**CTAGGATTGGTTACTGACGGTACC-3’) were subcloned into the *Kpn* I site of pGL4.26 (Promega, Madison, WI) with a minimal promoter driving the firefly luciferase reporter. C/EBP-β or c-MYC cDNA was amplified from total RNA of MCF-7 or HSC2 cells by RT-PCR and subcloned into pRc/CMV (Invitrogen, Carlsbad, CA) or pcDNA 3.1/V5-His-TOPO (Invitrogen). Constructs were confirmed by sequence analysis and designated as pGL4.26-EPAS1_G, pGL4.26-EPAS1_A, pCMV-C/EBP-β, or pcDNA-c-MYC, respectively. Rat c-Jun driven by the human β-actin promoter (c-Jun/β-actin) and human c-FOS expression vectors were generously provided by Dr. Masaharu Sakai (Hokkaido University) [31]. Transient transfections were performed where each pGL4.26-EPAS1 reporter construct (0.2 μg/15 mm well) with Renilla luciferase co-transfection control (pRL-SV40, 100 pg/15-mm well) (Promega) was mixed with 0.4 μl of Trans-IT LT1 Transfection Reagent (TaKaRa, Japan) and added to the medium. In co-transfection experiments, 0.4 μg of pRc/CMV, c-Jun/β-actin, c-FOS, pcDNA-c-MYC or pCMV-C/EBP-β was mixed with EPAS1 reporters as above. Cells were incubated with the mixture for 24 h prior to quantification of luciferase reporter activity on the single-sample Mini Lumat LB 9505 luminometer (Berthold Technologies GmbH & Co. KG, Bad Wildbad, Germany) using the Dual Luciferase Assay Kit (Promega). *EPAS1* SNP reporter activity was calculated as ratios over Renilla luciferase activity and the average of three assays or more for each reporter was used for comparisons.

### Patient studies

A total of 76 Japanese non-small cell lung cancer patients diagnosed at the Hiroshima University Hospital from 1991 to 1996 [4, 32] were enrolled in this study. Written informed consent was obtained from all individuals. This study was approved by the Institutional Genetic and Medical Ethics Committee at Hiroshima University. Patients and their clinicopathological characteristics of lung cancer were assessed according to the International Staging System for lung cancer [33], and are shown in Table 1. Briefly, a total of 76 lung cancer patients, whose average age was 65.8 (±8.2), consisted of 56 males and 20 female; 43 adenocarcinomas (AD), 29 squamous cell carcinomas (SCC), and 4 adeno-squamous cell carcinomas (ADSCC). Distribution of the lung cancer patients by stages (31 patients were at stage I, 7 at II, 25 at III and 13 at IV) is well matched to the broadly representative of the Japanese lung cancer population.

**Table 1:** Patient characteristics.

Patient demographics			
Total individual (%)		76 (100)	
Mean age (SD)		65.8	(8.2)
Gender (%)	Male	56 (73.7)	
	Female	20 (26.3)	
Differentiation^a^	Well	18 (25.0)	
	Moderate	35 (48.6)	
	Poor	19 (26.4)	
Stage^b^	I	31 (40.8)	
	II	7 (9.2)	
	III	25 (32.9)	
	IV	13 (17.1)	

^a^Differentiation was defined for NSCLC excluding four cases with adenosquamous cell carcinoma.

^b^Stage was assessed according to the International Staging System (Hermanek and Sobin, 1987).

### Statistical analysis

The primary outcomes in this study were lung cancer-specific mortality and risk of death. Survival time was calculated as the time from primary surgery to death due to lung cancer, censoring at the date of last contact or non-cancer death. Chi-square or Fisher’s exact test was used to examine distributions of variables. Differences in survival were examined using log-rank test. Cox proportional hazard model was used to estimate the relative risk for death and 95% confidence intervals (CI). Statistical analyses were performed using a statistical software JMP version 10.0.2 (SAS Institute Inc., Cary, NC, USA), otherwise specified. Extended Fisher's exact test was conducted using “R”: a language and environment for statistical computing (R Core Team, 2013. R Foundation for Statistical Computing, Vienna, Austria. URL http://www.R-project.org/) with a package “coin”. Hardy-Weinberg equilibrium was examined to compare the observed and expected genotype frequencies using a Chi-square test. Statistical significance of reporter experiments and gene expression analyses were analyzed using statistical softwares JMP version 10.0.2 and StatView version 5.0 software (SAS Institute Inc.).

## Results

### Database surveillance of SNPs of *EPAS1* in relation to its gene expression

Among the 3 Hap-tag SNPs (rs13419896, rs4953354, and rs4953388) that were implicated to contribute to the adaptation to high-altitude hypoxia in Sherpas [22], we found binding sites and binding activities for the C/EBP-β, AP-1 or MYC family of transcription factors in a number of cancer cell types in the region of the *EPAS1* rs13419896 locus within intron 1 of the gene by surveillance of ChIP-seq datasets from the ENCODE (S1 Fig). Interestingly enough, when analyzed the sequences using JASPAR Core Vertebrata, we found that relative scores, indices for probability of transcription factor binding, of C/EBP-β and AP-1 were affected by the rs13419896 SNP among the 3 transcription factors as mentioned above; C/EBP-β and AP-1 showed much higher scores of 0.842 and 0.855 in the sequence with *A* allele at rs13419896, respectively, than those with *G* allele (0.734 and 0.744). These data prompted us to examine the role of rs13419896 in regulation of the *EPAS1* gene expression.

### The rs13419896 SNP altered the reporter gene activities

**Fig 1:**
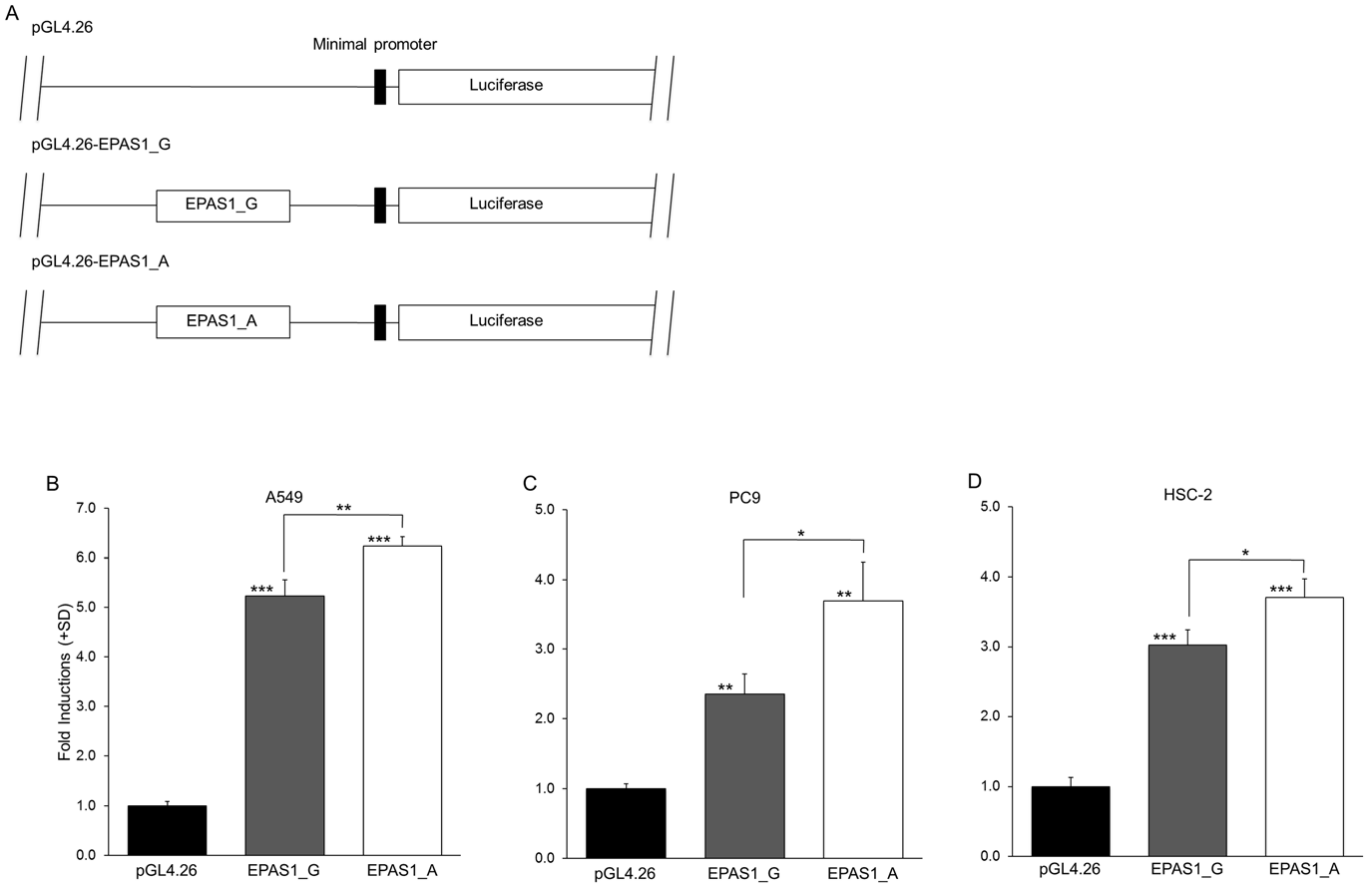
*EPAS1* SNP luciferase reporter activities in cancer cell lines. (A) Schematic representation of luciferase reporter constructs with different *EPAS1* SNP alleles as shown. Short fragments containing the *EPAS1* SNP locus (rs13419896) were subcloned into the pGL4.26 minimal promoter luciferase reporter plasmid. (B-D) Bar charts show luciferase reporter activity after transient transfection experiments in A549 (B), PC9 (C), and HSC-2 (D) cells. Luciferase reporter activity was calculated as a ratio to Renilla luciferase activity generated by the pRL-SV40 co-transfection control. Each value represents the mean + standard deviation (SD) for at least three independent experiments. *P*-values were calculated using Student’s *t*-test with *: *P* < 0.05, **: *P* < 0.01 and ***: *P* < 0.0001.

In order to test possible functional differences caused by the rs13419896 locus, we next prepared luciferase reporter constructs harboring the *EPAS1* fragment encompassing the rs13419896 locus in front of the minimum promoter (Fig 1A) and performed transient transfection analyses in A549, PC-9 and HSC-2 cancer cell lines. The constructs with the fragments containing the rs13419896 locus showed increased reporter activities as compared to the original reporter pGL4.26 in all the cell lines tested, regardless of the genotype of the SNP, suggesting that this short sequence within the intron 1 of *EPAS1* function as a transcriptional enhancer element (*P* < 0.05, Fig 1B–1D). Interestingly, the reporter construct containing the fragment with the *A* allele of rs13419896 (pGL4.26-EPAS1-A) showed significantly higher activity than one with the *G* allele (pGL4.26-EPAS1-G) in all the cell lines tested (*P* < 0.01, *P* < 0.05 as shown in Fig 1B–1D), suggesting that the enhancer activity of this regulatory element is affected by the genotype of the rs13419896 SNP.

**Fig 2:**
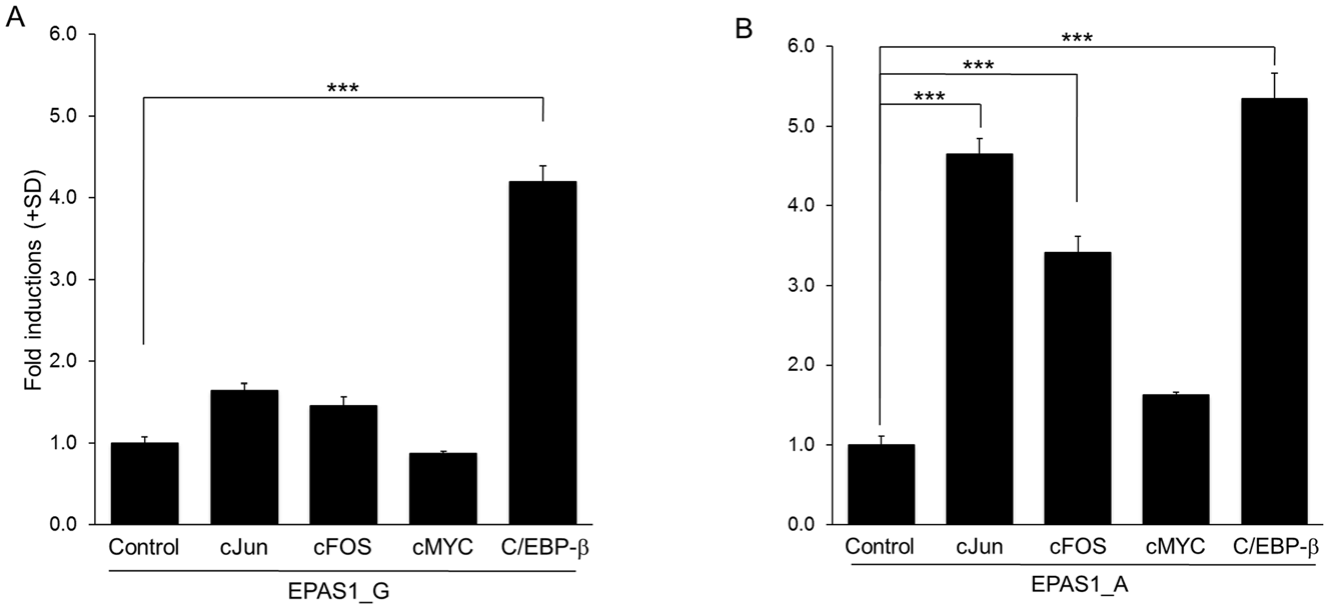
Effects of database-suggested transcription factors on rs13419896 SNP luciferase reporter activities. (A and B) To evaluate the effects of overexpressing AP-1 (c-Jun and c-FOS), MYC, or C/EBP-β transcription factors on rs13419896 SNP luciferase reporter activity, co-transfection experiments were performed in A549 lung adenocarcinoma cells. Bar charts show luciferase reporter activity of pGL4.26-EPAS1-G (A) or pGL4.26-EPAS1-A (B). Luciferase reporter activity was calculated as a ratio to Renilla luciferase activity generated by the pRL-SV40 co-transfection control. Each value represents the mean + standard deviation (SD) for at least three independent experiments. *P*-values were calculated using Dunnett's test with ***: *P* < 0.0001.

Since the rs13419896 SNP was implicated to alter binding affinities of AP-1 and C/EBP-β by the aforementioned bioinformatics analysis, we next performed co-transfection experiments in A549 cells to evaluate the effects of overexpressing AP-1, c-MYC, or C/EBP-β transcription factors on the reporter activities. Surprisingly, forced expression of c-Jun or c-FOS, components of the transcription factor AP-1, significantly increased the reporter activity of only pGL4.26-EPAS1-A but not that of pGL4.26-EPAS1–G (Fig 2A and 2B). On the other hand, C/EBP-β increased the transcriptional activity of both of the reporter constructs with similar amplitude, while c-Myc did not show significant alterations in the activity of both constructs.

### Comparison of expression levels of *EPAS1* mRNA and protein in cancer cell lines by the rs13419896 SNP

**Fig 3:**
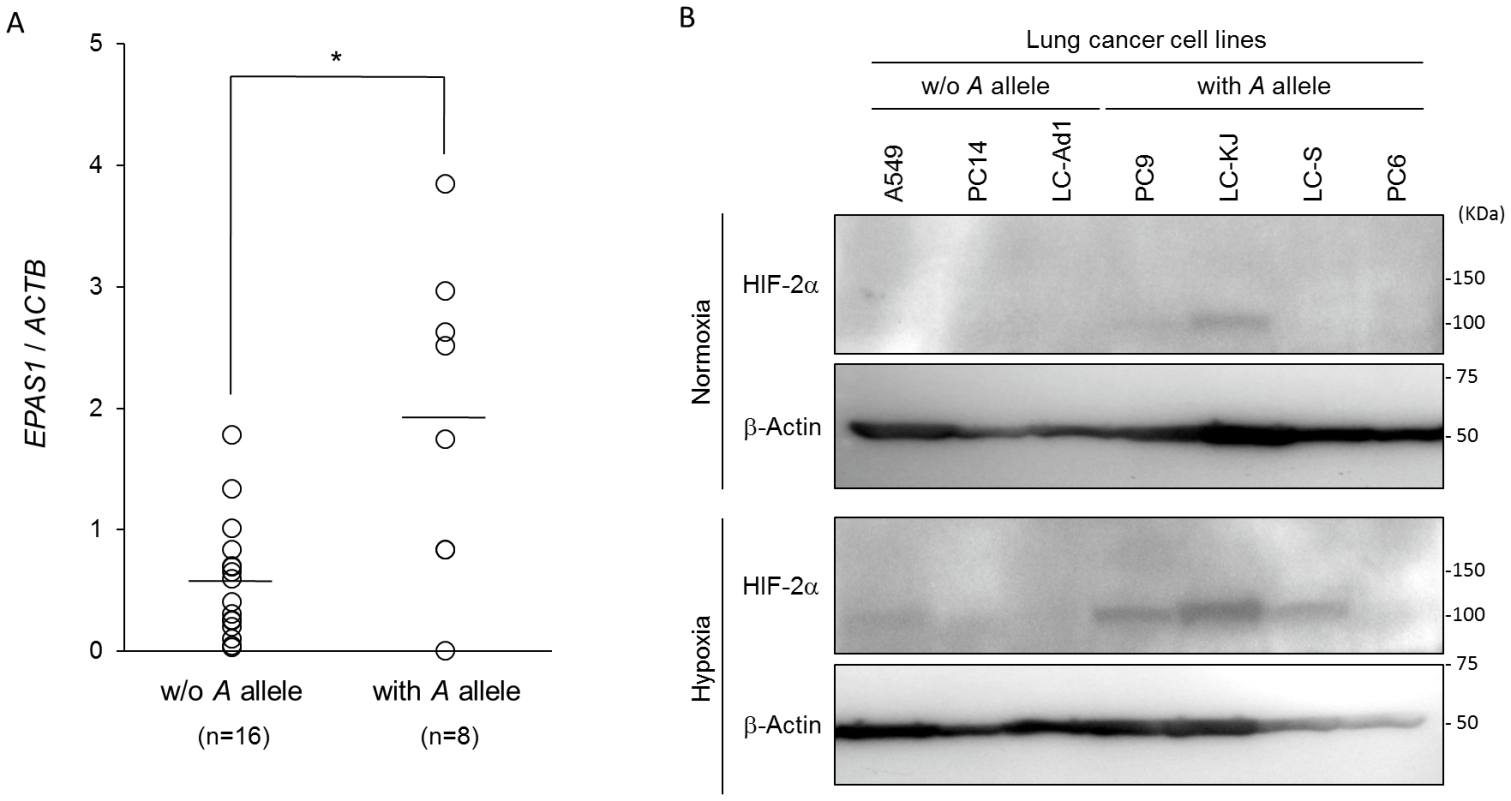
Expression levels of *EPAS1* mRNA in cancer cell lines by the rs13419896 SNP. (A) The *EPAS1* gene expression levels evaluated by real-time RT-PCR were compared between cancer cells’ groups by rs13419896 status; one with the *A* allele at the SNP site (with *A* allele) and others without (w/o *A* allele). The expression levels of *EPAS1* in each cell line was calculated as a ratio to that of *ACTB* and each value represents the mean for at least three independent experiments (open circle). Each bar indicates average of expression level in each group. *P*-values were calculated using Welch’s *t* test with *: *P* = 0.022. (B) The EPAS1 protein expression levels were compared between genotypes as above. Several lung cancer cell lines were incubated under normoxic (21% pO_2_) or hypoxic (1% pO_2_) for 24 hours. Whole cell extracts prepared from each cell line were subjected in immunoblotting analysis using anti-EPAS1 (HIF-2α) or anti-β-actin as a control.

In order to explorer effects of the rs13419896 SNP on endogenous gene expression levels of the *EPAS1*, we genotyped the SNP and evaluated levels of the gene expression in diverse cancer cell lines. When the cells were divided into two groups by presence or absence of the *A* allele at the rs13419896 locus, cancer cells with the *A* allele of the rs13419896 demonstrated significantly higher *EPAS1* gene expression levels than for any others without the *A* allele (*P* = 0.022, Welch’s *t* test)(Fig 3A). We further analyzed levels of EPAS1 (HIF-2α) protein expression in several lung cancer cell lines by immunoblotting analyses. As results, EPAS1 proteins were detected in PC9 and LC-KJ with the *A* allels even under normoxic conditions and were obviously increased in some of hypoxic cancer cells, A549, PC9, LC-KJ, and LC-S (Fig 3B).

### The A allele of rs13419896 SNP of *EPAS1* was associated with poor overall survival of non-small cell lung cancer patients

Since the rs13419896 SNP was suggested to link with enhanced expression of *EPAS1* gene, we then explored possible associations of the SNP with clinicopathological factors of Japanese NSCLC patients. Genotypes of the rs13419896 SNP were determined in 76 NSCLC patients, and found to be in good agreement with Hardy-Weinberg equilibrium (*P* = 0.45, Chi-square test, Table 2). No significant difference was found amongst genotype frequencies between the Japanese NSCLC samples in this study and the healthy Japanese population of the HapMap project (*P* = 0.94, extended Fisher’s exact test, Table 2). We then examined the relationship between the *EPAS1* SNPs and various clinicopathological characteristics (Table 3). The frequency of minor allele of rs13419896 tended to be higher in females than in males: A frequency of patients possessing at least one *A* allele of rs13419896 (*A*/*A* or *A*/*G* genotype) were 70.0% (14 of 20) in females and 44.6% (25 of 56) in males, though this was not statistically significant (*P* = 0.07, Fisher’s exact test). In addition, distribution of differentiation tended to differ by the SNP with a marginal significance (*P* = 0.06, extended Fisher’s exact test). Other than the gender and differentiation, we did not find any statistical associations of the SNP with clinicopathological characteristics including age, histology, tumor size, and stage.

**Table 2:** Genotype frequencies of the *EPAS1* rs13419896 SNP in Japanese NSCLC patients with those in HapMap-JPT.

Genotype	NSCLC		HapMap-JPT^a^		*P* ^b^
	n	%	n	%	
*G/G*	37	48.7	52	46.0	0.94
*A/G*	30	39.5	46	40.7	
*A/A*	9	11.8	15	13.3	
*G* allele	104	68.4	150	66.4	0.74
*A* allele	48	31.6	76	33.6	

^a^ http://hapmap.ncbi.nlm.nih.gov/cgi-perl/snp_details_phase3?name=rs13419896&source=hapmap28_B36&tmpl=snp_details_phase3

^b^(Extended) Fisher’s exact test

**Table 3:** Associations of various clinicopathological factors with *EPAS1* rs13419896 polymorphism in NSCLC patients.

		rs13419896		
		*G/G*	*A/G* or *A/A*	*P*
Mean Age (yr)		65.0	66.6	0.41
(SD)		7.7	8.6	
Gender (n)	Male (%)	31 (40.8)	25 (32.9)	0.07
	Female (%)	6 (7.9)	14 (18.4)	
Histology (n)	Adenocarcinoma (%)	20 (26.3)	23 (30.3)	0.93
	Adenosquamous Carcinoma (%)	2 (2.6)	2 (2.6)	
	Squamous Cell Carcinoma (%)	15 (19.8)	14 (18.4)	
Stage (n)	I (%)	16 (21.1)	15 (19.7)	0.13
	II (%)	6 (7.9)	1 (1.3)	
	III (%)	11 (14.5)	14 (18.4)	
	IV (%)	4 (5.3)	9 (11.8)	
Differentiation (n)	Well (%)	5 (6.6)	13 (17.1)	0.06
	Moderately (%)	16 (21.0)	19 (25.0)	
	Poorly (%)	13 (17.1)	6 (7.9)	
	NA^b^ (%)	3 (4.0)	1 (1.3)	
Mean tumor size^a^ (cm)		3.9	4.4	0.20
(SD)		1.5	1.7	

^a^Two cases were missing for the data of tumor size.

^b^NA, not applicable.

**Fig 4:**
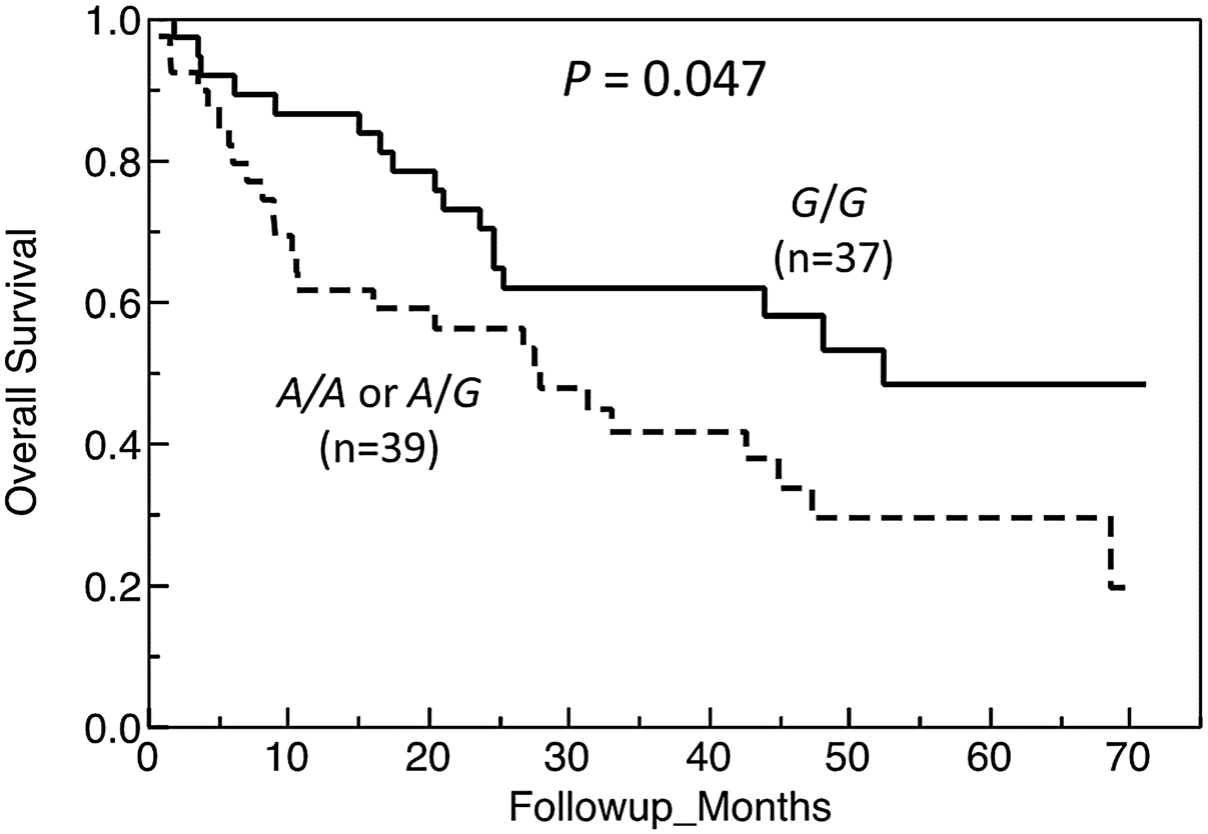
Overall survival of NSCLC patients by the rs13419896 SNP in *EPAS1*. Kaplan-Meier survival plots stratified according to genotypes of rs13419896 are shown. Difference in overall survival across genotypic groups of NSCLC patients was examined using the log-rank test with *P*-values as indicated.

We then assessed association of the rs13419896 SNP with overall survival for the NSCLC. The median survival time of patients with at least one *A* allele of rs13419896 (*A*/*A* or *A*/*G*) was significantly shorter than that with the *G*/*G* homozygote (28.0 months vs. 52.5 months, *P* = 0.047, log-rank test, Fig 4). When compared cumulative survival rates at 12, 24, and 48 months, patients with the *G/G* homozygote showed much higher rates than those with *A/G* or *A/A* genotype at 12 and 48 months (*P* = 0.009 and 0.004, respectively) (S1 Table). A multivariate analysis of the 74 NSCLC patients (2 patients were excluded because of the lack of tumor size data) using a Cox proportional hazard model demonstrated that the possession of *A* allele (*A/G* or *A/A* genotype) of rs13419896, along with clinical stage, was an independent variable for risk estimation of overall survival for NSCLC patients [hazard ratio (HR) = 2.31, 95% CI = 1.14–4.81, *P* = 0.021], after adjustment for age, gender, stage, histology, tumor size, and differentiation.

## Discussion

Recently, several SNPs of *EPAS1* have been shown to correlate with the development of various diseases such as osteoarthritis [16], retinopathy of prematurity [17], maximum metabolic power in elite endurance athletes [18], physiologic adaptation in high altitude populations [19–22], and susceptibility towards renal cell carcinoma (RCC) and prostate cancer [23, 24]. However, a mechanistic link between these SNPs and gene expression levels of the *EPAS1* has scarcely been known, except for the rs17039192 located in the 5’-untranslated region, that gave altered promoter activities in reporter gene assay in chondrogenic cells [16].

In this study, bioinformatic analyses prompted us to test a role of one of the Hap-tag SNPs, rs13419896 located within intron1 of the gene, in regulation of *EPAS1* expression. In fact, we found that a fragment in the intron 1 of *EPAS1* contains transcriptional regulatory elements and nucleotide difference at the rs13419896 locus may functionally affect enhancer activities in cancer cell lines. Interestingly, further co-transfection experiments strongly indicated that AP-1 transcription factor might be involved in the differential transcriptional activities between the rs13419896 alleles. The observed specific transactivation by exogenous AP-1 components only in constructs with *A* allele at the rs13419896 agreed well with the higher relative score for AP-1 with *A* allele at the SNP as shown by JASPAR Core Vertebrata analysis. Previously, overexpression of c-Jun and c-Fos proteins was observed in 31–50 and 60%, respectively, of NSCLC tissues [34, 35]. The overexpressed c-Jun or c-Fos may transactivate the *EPAS1* gene expression *via*, at least in part, an enhancer element within intron 1 of the gene in an allele-specific manner at the rs13419896 locus. This was also supported by the observation of gene and protein expression levels of *EPAS1* by the rs13419896 SNP in various cancer cells. Although we did not completely confirm the genotypes (including allelic loss, amplification, or mutations) of the cancer cell lines tested, we found that the cells with *A* allele at rs13419896 of *EPAS1* showed significantly higher *EPAS1* gene and protein expression levels compared with those lacking *A* allele regardless of differences in genetic background. Taken together these data, it is strongly suggested that the *A* allele at rs13419896 SNP of *EPAS1* plays an important role in alteration of binding affinity of AP-1, resulting in differentiated levels of expression of the EPAS1 in NSCLC tissue.

The observed association of the *A* allele of the rs13419896 SNP with increased expression levels of EPAS1 inspired us to further examine the possible role of the SNP in prognosis of Japanese NSCLC patients, since overexpression of EPAS1 was reported to be associated with a poor prognosis. We demonstrated for the first time that the rs13419896 locus was an independent variable for risk estimation of overall survival of NSCLC.

In human NSCLC, overexpression of HIF-2α (EPAS1) was consistently associated with histology as SCC being dominant, increased tumor size, and angiogenesis, resulting in worse prognosis and decreased survival rates [13, 14]. In our study, we did not find any association of the rs13419896 SNP with histology and tumor size. On the other hand, the hazard ratio of possession of *A* allele over the *G* allele in Cox's hazard model was 2.31, that is comparable to the ratio of high expression of HIF-2α obtained previously (2.01 and 1.71) [13, 14]. Recent meta-analysis examining overall survival by the overexpression of HIF-2α protein also demonstrated HR of 2.02 (95%CI: 1.47–2.77) [36]. Considering these, the rs13419896 SNP may be one of the important factors that contribute to the overexpression of HIF-2α in NSCLC tissue and thus be a useful prognostic marker for NSCLC. We observed a statistically significant difference of cumulative survival rate between patients with *A* allele and without at 12 months post operation (S1 Table). If our observation is confirmed by other cohorts in future, genotyping of the SNP may become clinically important for considering patients’ care and counseling immediately. Besides NSCLC, other cancers such as colorectal and head and neck cancers were also reported to show a poor prognosis with overexpression of the HIF-2α in meta-analyses [37, 38]. Since overexpression of AP-1 components can be observed for these cancers [39, 40], the rs13419896 SNP may contribute to the overexpression of HIF-2α and be a useful prognostic marker in various cancers.

In conclusion, we found here for the first time that nucleotide difference at the rs13419896 SNP may affect *EPAS1* gene and protein expression, specifically in response to AP-1, and that the *A* allele of *EPAS1* SNP is associated with poorer prognosis of lung cancer patients. To establish the *EPAS1* SNP as a useful clinical prognostic marker and to further clarify their molecular mechanisms, larger scale clinicopathological studies of lung cancer and/or other types of cancer will provide additional insights into these aspects.

## Supporting Information

S1 Fig UCSC Genome Browser representation of ENCODE Consortium ChIP-Seq data for transcription factor binding overlaid on human genome build hg19. Top panels show genomic structure of the *EPAS1* gene compiled from UCSC, RefSeq and GenBank with thick bars indicating exonic coding sequences, thin bars showing non-coding exon regions (5’ and 3’ UTRs) and arrows denoting introns with 5’ to 3’ directionality. The horizontal axis shows genome position in bp in the interval from chr2: 46,514,938–46,626,784. The position of the rs13419896 SNP is indicated in red and its relative position is extrapolated across all datasets as a broken blue line. ChIP-Seq data is shown in the same genome location with the vertical axis indicating ChIP enrichment of transcription factor binding for CEBPB (black), MYC (red), FOS (green), JUN (blue), JUNB (violet) and JUND (orange) in the specified cell lines. Scale bar indicates genomic distance of 50 kb.
(PDF)

S1 Table Comparisons of cumulative survival rates between patients with genotypes *G*/*G* and *A*/*G* or *A*/*A*. (DOCX)
