# Supplementary figures and images for: The A Allele at rs13419896 of EPAS1 Is Associated with Enhanced Expression and Poor Prognosis for Non-Small Cell Lung Cancer
Source: PLoS One. 2015 Aug 11;10(8):e0134496. doi: 10.1371/journal.pone.0134496 (PMC4532412; doi:10.1371/journal.pone.0134496)

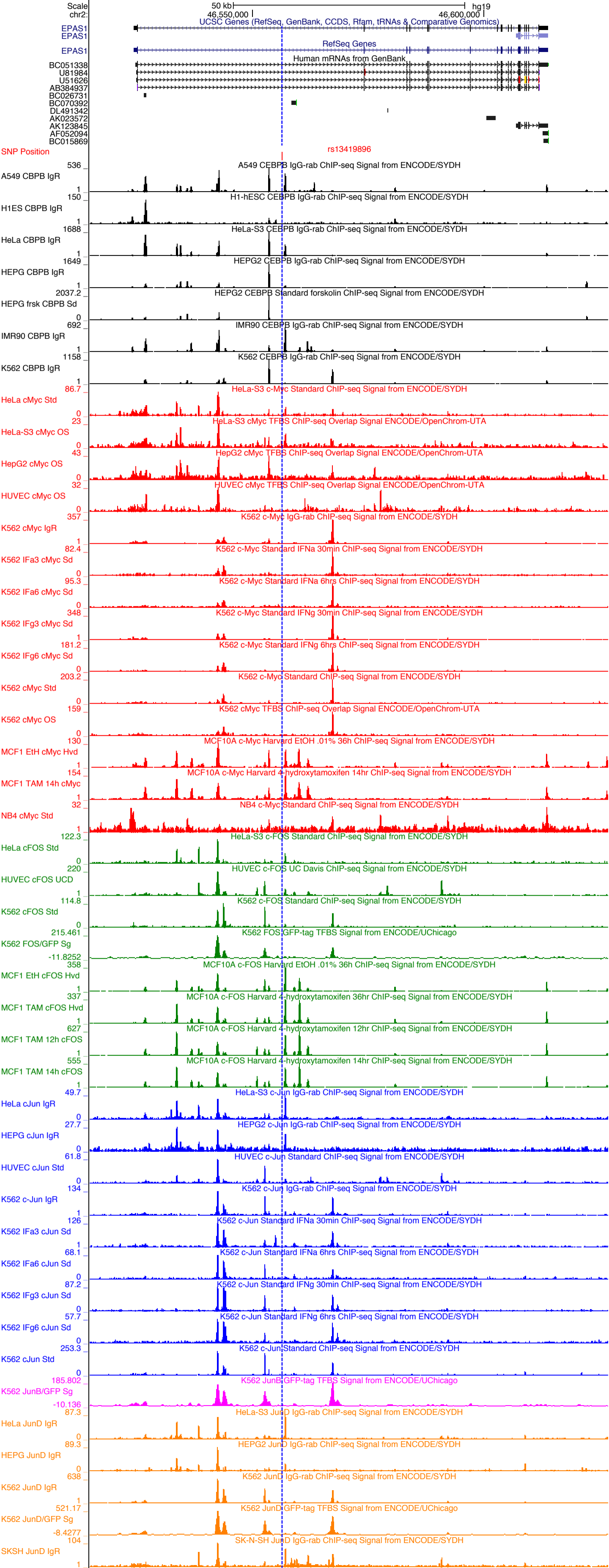

Supplement: S1 Fig — Top panels show genomic structure of the EPAS1 gene compiled from UCSC, RefSeq and GenBank with thick bars indicating exonic coding sequences, thin bars showing non-coding exon regions (5’ and 3’ UTRs) and arrows denoting introns with 5’ to 3’ directionality. The horizontal axis shows genome position in bp in the interval from chr2: 46,514,938–46,626,784. The position of the rs13419896 SNP is indicated in red and its relative position is extrapolated across all datasets as a broken blue line. ChIP-Seq data is shown in the same genome location with the vertical axis indicating ChIP enrichment of transcription factor binding for CEBPB (black), MYC (red), FOS (green), JUN (blue), JUNB (violet) and JUND (orange) in the specified cell lines. Scale bar indicates genomic distance of 50 kb. (PDF) [file pone.0134496.s001.pdf]
