# Supplementary material for: The A Allele at rs13419896 of EPAS1 Is Associated with Enhanced Expression and Poor Prognosis for Non-Small Cell Lung Cancer
Source: PLoS One. 2015 Aug 11;10(8):e0134496. doi: 10.1371/journal.pone.0134496 (PMC4532412; doi:10.1371/journal.pone.0134496)
Supplement: S1 Table — (DOCX) [file pone.0134496.s002.docx]

**Supporting Table S1** Comparisons of cumulative survival rates between patients with genotypes *G*/*G* and *A*/*G* or *A*/*A*.

|  | *G*/*G* | |  | *A*/*G* or *A*/*A* | |  |  |
| --- | --- | --- | --- | --- | --- | --- | --- |
| Months | Cumulative survival rate | SE |  | Cumulative survival rate | SE |  | *P* |
| 12 | 0.8649 | 0.0562 |  | 0.6154 | 0.0779 |  | 0.009 |
| 24 | 0.7027 | 0.0751 |  | 0.5617 | 0.0799 |  | 0.197 |
| 48 | 0.5797 | 0.0841 |  | 0.2339 | 0.0829 |  | 0.003 |

SE, standard error. Statistical significance was evaluated by two-tailed.
